# Development at Walsall General Hospital

**Published:** 1920-04-10

**Authors:** 


### Development at Walsall General Hospital.

The development of Walsall General Hospital during the last few years has been very marked. Although containing only ninety beds, it compares favourably with many larger hospitals in its scope and aims. Its special departments now comprise: (*a*) An orthopædic department, with complete set of remedial and gymnastic apparatus; (*b*) a massage and electrical department, with electric baths, radiant-heat baths, plinths, etc.; (*c*) an $x$-ray department (this is now to be enlarged and provided with the most modern apparatus); (*d*) a fully-equipped and largely attended department for the treatment of venereal disease; (*e*) special eye, ear, nose, and throat departments for the treatment of school children.

Other features are the special treatment of malaria; the treatment of nævi, etc., by carbon-dioxide snow; psycho-therapeutic treatment; ionisation treatment, etc.; and it is hoped soon to include the special study of tropical diseases. In 1915 the ordinary income was £4,811; last year it was £10,090. During the last few months the Committee have added a complete and well-furnished night nurses' home and a fully-equipped pathological laboratory. They are now building a diphtheria ward. The present site is unfortunately so small that a much-needed extension is impossible there, and the Committee are now purchasing a large piece of land on the outskirts of Walsall, where a new hospital can be built when funds permit.

